# Influence of dronedarone (a class III antiarrhythmic drug) on the anticonvulsant potency of four classical antiepileptic drugs in the tonic–clonic seizure model in mice

**DOI:** 10.1007/s00702-018-1940-y

**Published:** 2018-12-08

**Authors:** Katarzyna M. Sawicka, Agnieszka Wawryniuk, Jadwiga Daniluk, Sławomir Karwan, Magdalena Florek-Łuszczki, Jarosław Chmielewski, Jarogniew J. Łuszczki

**Affiliations:** 1 Department of Internal Medicine in Nursing, Medical University of Lublin, Lublin, Poland; 2 Department of Pathophysiology, Medical University of Lublin, Lublin, Poland; 3 Pope John Paul II State School of Higher Education in Biala Podlaska, Biala Podlaska, Poland; 4 Regional Specialized Children’s Hospital, Olsztyn, Poland; 5 Department of Medical Anthropology, Institute of Rural Health, Lublin, Poland; 6 Institute of Environmental Protection-National Research Institute in Warsaw, Warsaw, Poland; 7 Isobolographic Analysis Laboratory, Institute of Rural Health, Lublin, Poland

**Keywords:** Dronedarone, Antiarrhythmic drugs, Antiepileptic drugs, Drug interactions

## Abstract

Increasing evidence indicates that some antiarrhythmic drugs play a pivotal role in seizures, not only in vivo studies on animals, but also in clinical trials. Some of these antiarrhythmic drugs potentiate or alleviate the anticonvulsant action of the classical antiepileptic drugs. The aim of this study was to determine the influence of dronedarone (DRO—a multichannel blocker belonging to the class III of antiarrhythmic drugs) on the anticonvulsant effects of four standard antiepileptic drugs (carbamazepine, phenobarbital, phenytoin and valproate) in the tonic–clonic seizure model in mice. Potential acute adverse effects exerted by the antiepileptic drugs combined with DRO were evaluated in three behavioral tests (chimney, grip-strength and passive avoidance). To confirm the nature of interaction, total brain concentrations of antiepileptic drugs were measured. DRO (50 mg/kg, i.p.) significantly reduces the anticonvulsant potency of phenytoin (*P* < 0.05), having no impact on that of carbamazepine, phenobarbital and valproate in the tonic–clonic seizure model in mice. DRO (50 mg/kg) neither changed total brain concentrations of phenytoin in mice, nor affected normal behavior in experimental animals subjected to the chimney, grip-strength and passive avoidance tests. In conclusion, DRO should not be combined with phenytoin because it reduced the anticonvulsant effects of the latter drug in experimental animals. The combined administration of DRO with carbamazepine, phenobarbital and valproate resulted in neutral interaction between these drugs in the tonic–clonic seizure model in mice.

**Electronic supplementary material:**

The online version of this article (10.1007/s00702-018-1940-y) contains supplementary material, which is available to authorized users.

## Introduction

According to World Health Organization, cardiovascular diseases are still the most frequent cause of death and disability in humans in both, developed and developing countries (Lee et al. 2017; Saner and van der Velde 2016). Prevention and treatment of these diseases is a principal goal of public health policies in many countries (Shantsila and Lip 2013; Varma and Ricci 2013). To reduce number of patients with cardiovascular diseases and heart problems, some preventive initiatives are undertaken, including good nutrition and healthy life style. Additionally, some drugs are recommended to reduce mortality in patients with severe heart diseases (Baroletti et al. 2010; Gatzoulis et al. 2017; Steinberg et al. 2016).

Relatively recently, dronedarone (DRO—a novel antiarrhythmic drug) has been licensed to the treatment of heart diseases. This drug is a multichannel blocker because it blocks the rapidly and slowly activating delayed-rectifier potassium currents (*I*Kr, *I*Ks), the inward rectifier potassium currents (*I*K1), the acetylcholine activated potassium currents (*I*K, ACh), sodium currents (*I*Na) and L-type calcium currents (*I*CaL) (Patel et al. 2009). Dronedarone exhibits also anti-adrenergic effects, belonging to the class III antiarrhythmic drugs (Patel et al. 2009). Principal indications for DRO are atrial fibrillation and atrial flutter in patients, who underwent direct current cardioversion to maintain normal heart rhythm, as an alternative to amiodarone (Deneer and van Hemel 2011).

Accumulating evidence indicates that antiarrhythmic drugs also play an essential role in epilepsy patients. Previously, it has been documented that amiodarone, ivabradine, sotalol and propafenone (various anti-arrhythmic drugs) elevated the threshold for electroconvulsions and influenced the anticonvulsant properties of the selected classical antiepileptic drugs in the maximal electroshock-induced seizure test—an experimental model of tonic–clonic seizures (Banach et al. 2016, 2017, 2018; Luszczki et al. 2013; Sawicka et al. 2017).

Existence of two or more various comorbidities in one patient, especially, atrial fibrillation and epilepsy, especially, in elderly people is very likely. In such a situation, dronedarone can be administered together with antiepileptic drugs to prevent heart diseases and seizures. However, less is known about possible interaction between drugs, therefore, we sought to assess the anticonvulsant potency of four classical antiepileptic drugs, namely, carbamazepine, phenobarbital, phenytoin and valproate in the model of tonic–clonic seizures in male albino Swiss mice.

## Materials and methods

### Animals

In this study, we used adult male albino Swiss outbred mice (8-week-old, weighing 24 ± 3 g). After adaptation to laboratory conditions, the mice were randomly assigned to experimental groups comprising eight mice per group. Experiments complied with the ARRIVE guidelines and were conducted in strict accordance with the EU Directive 2010/63/EU for animal experiments. Total number of mice used in this study was 464.

### Drugs

DRO (Multaq^®^, Sanofi-Aventis, Paris, France), phenobarbital (Polfa, Krakow, Poland), carbamazepine, phenytoin and valproate (all three antiepileptic drugs from Sigma-Aldrich, Poznan, Poland) were used in this study. Only valproate (magnesium salt) was dissolved in sterile distilled water, whereas the other drugs were suspended in a 1% aqueous solution of Tween 80 (Sigma-Aldrich, Poznan, Poland). The drugs were administered intraperitoneally (i.p.): carbamazepine and valproate—30 min, phenobarbital—60 min, and DRO and phenytoin—120 min before electrically evoked seizures, all experiments evaluating behavior in animals and collection of brain tissues for measurement of antiepileptic drug content. Information about the treatment times and route of drug administration was from the literature and our previous experiments (Kondrat-Wrobel and Luszczki 2016; Sawicka et al. 2017; Zolkowska et al. 2016). All experiments were conducted in a blinded manner.

### Threshold for tonic–clonic seizures

The threshold for tonic–clonic seizures was used to determine the anticonvulsant properties of DRO when administered separately. An alternating current (50 Hz; 500 V) generated by rodent shocker (Hugo Sachs Elektronik, Freiburg, Germany), delivered via ear-clip electrodes, evoked tonic–clonic seizures in experimental animals. Duration of electric stimulus was 0.2 s and tonic hind limb extension in the mice was the endpoint in this seizure model. To determine the threshold for electroconvulsions in control animals, five groups of vehicle-treated animals were subjected to currents of increasing intensities from 5 to 9 mA. The electroconvulsive threshold was expressed as the median current strength value (CS_50_ in mA) necessary to evoke tonic hind limb extension in 50% of the tested animals. This procedure was also used to determine the seizure threshold for animals receiving increasing doses of DRO (50, 75 and 100 mg/kg). In such a situation, the current intensities applied to determine the threshold for DRO ranged from 7 to 11 mA. The logit-probit method was used to calculate CS_50_ values (Litchfield and Wilcoxon 1949). Total number of mice used in this procedure was 128.

### Tonic–clonic seizures in mice

The anticonvulsant effects of four classical antiepileptic drugs administered either alone or in combination with DRO were determined in the tonic–clonic seizure model in mice. An alternating current (25 mA; 50 Hz; 500 V) generated by a rodent shocker (Hugo Sachs Elektronik, Freiburg, Germany), delivered via ear-clip electrodes, evoked seizure activity in mice. Duration of electric stimulus was 0.2 s and the tonic hind limb extension was established as the endpoint. At least 3 groups of the mice, received increasing doses of the classical antiepileptic drugs (either alone or in combination with DRO), and were subjected to the current stimulation. Protective effects observed in animals challenged with the electroconvulsions (tonic–clonic seizures) were expressed as median effective doses (ED_50_) of the anticonvulsant drug, as described earlier (Litchfield and Wilcoxon 1949). Of note, the ED_50_ value corresponds to a dose of an anticonvulsant drug (in mg/kg), which protects 50% of the mice from the tonic–clonic seizures. In this study, we determined the ED_50_ values for the classical anticonvulsant drugs administered alone and for their combination with DRO (administered in a dose of 50 mg/kg). When a significant change in the ED_50_ value for the classical antiepileptic drug was observed, we evaluated the combination of that drug with a reduced dose of DRO (25 mg/kg) to exclude any non-specific interaction between drugs in the tonic–clonic seizure model. This was the reason to determine the anticonvulsant effects for the combination of phenytoin with DRO (25 mg/kg). Total number of animals used in this procedure was 240.

### Grip-strength test

The effects of DRO administered alone, antiepileptic drugs administered alone and their combinations (in doses reflecting their ED_50_ values from the tonic–clonic seizure model), on muscular strength of forelegs in mice were determined with the grip-strength test, as described elsewhere (Meyer et al. 1979; Zadrozniak et al. 2009). Skeletal muscular strength in experimental animals, after receiving the respective treatment, was measured with electronic dynamometer and expressed in newton (N) as means ± S.E.M. of 8 determinations. Total number of mice used in this procedure was 80.

### Chimney test

The effects of DRO administered alone, antiepileptic drugs administered alone and their combinations (in doses reflecting their ED_50_ values from the tonic–clonic seizure model), on motor coordination in mice were determined with the chimney test, as described elsewhere (Boissier et al. 1960). Motor coordination in experimental animals, after receiving the respective treatment, was estimated by researchers, who placed each mouse individually in the plastic transparent tube (3 cm inner diameter, 30 cm long) positioned vertically on the table. Every mouse had to climb backwards up the tube within 1 min. The mice that failed to escape the tube within 60 s displayed impairment of motor coordination and were classified as animals with motor deficits. Total number of mice used in this procedure was 80.

### Step-through passive avoidance task

The effects of DRO administered alone, antiepileptic drugs administered alone and their combinations (in doses reflecting their ED_50_ values from the tonic–clonic seizure model), on long-term memory (acquisition, learning and remembering) in mice were determined with passive avoidance task, as described in details elsewhere (Luszczki et al. 2005; Venault et al. 1986). Briefly, on the first day of experimentation, the animals after receiving drugs either alone or in combination were placed in an illuminated box (10 × 13 × 15 cm) connected to a larger dark box (25 × 20 × 15 cm) equipped with an electric grid floor. When the animal enters the dark box, a guillotined door between these two boxes was shut and an electric footshock (0.6 mA for 2 s) was delivered via electric grid floor. On the following day (24 h later), the pre-trained mice were placed again into the illuminated box and observed up to 180 s. The animals that avoided the dark compartment for 180 s were considered to remember the task. The time that the mice took to enter the dark box was noted. The median retention times (in seconds with 25th and 75th percentiles) of 8 determinations indicated long-term memory in mice. Total number of mice used in this experimental procedure was 80.

### Measurement of total brain antiepileptic drug concentrations

In this study, only total brain concentrations of phenytoin co-administered with DRO (50 mg/kg) were measured because the anticonvulsant effect of this anticonvulsant drug significantly differed from those for control (phenytoin + vehicle-treated) mice. Pharmacokinetic measurement of total brain concentrations of phenytoin was performed using fluorescence polarization immunoassay technique. To measure phenytoin content in brain tissues of experimental animals, the mice after injecting with phenytoin alone and in combination with DRO were decapitated (at times that coincided with the pretreatment times from the tonic–clonic seizure test). Next, the whole brains of the mice were removed from skulls, weighed, harvested and homogenized using Abbott buffer (1:2 weight/volume), as described elsewhere (Luszczki et al. 2006, 2018). After centrifugation of the homogenates (at 10,000*g* for 10 min), 200 µl of supernatant samples were transferred to an Architect 4000 analyzer (Abbott) using manufacturer supplied kits for the measurement of phenytoin content. Concentrations of phenytoin in the brain of experimental animals were expressed in µg/ml of brain supernatants as means ± S.E.M. of eight separate brain preparations. Total number of mice used in this procedure was 16.

### Statistical analysis

The CS_50_ values for DRO administered alone and the ED_50_ values for antiepileptic drugs administered alone or in combination with DRO were calculated using log-probit method (Litchfield and Wilcoxon 1949), as described in detail earlier (Luszczki et al. 2009). Multiple comparisons of CS_50_ values (± S.E.M.) from the threshold test and ED_50_ values (± S.E.M.) from the tonic–clonic seizure model were performed using one-way analysis of variance (ANOVA) followed by the post-hoc Tukey–Kramer test. Single comparisons between the ED_50_ values (± S.E.M.) were statistically analyzed with log-probit method (Litchfield and Wilcoxon 1949). Total brain concentrations of phenytoin were statistically verified with unpaired Student’s *t* test. Mean muscular strengths (± S.E.M.) from the grip-strength test were analyzed with one-way ANOVA accompanied with the Holm–Sidak test. The Fisher’s exact probability test was used to statistically compare the results from the chimney test. Non-parametric Kruskal–Wallis test was used to statistically verify the results from the passive avoidance task. Statistical significance was set up at *P* < 0.05. To analyze the data, GraphPad Prism version 7.0 for Windows (GraphPad Software, San Diego, CA, USA) was used. All statistical tests (i.e., Tukey–Kramer, Holm–Sidak, Fisher, Kruskal–Wallis) were automatically computed by GraphPad Prism version 7.0.

## Results

### Effect of dronedarone on the threshold for maximal electroconvulsions in mice

**Fig. 1:**
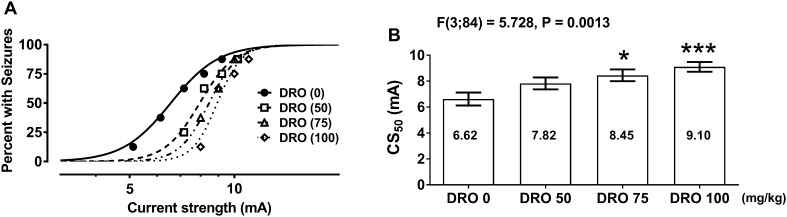
Influence of dronedarone (DRO) on the threshold for maximal electroconvulsions in mice. Left panel graph illustrates current intensity–response function for tonic hind limb extension in the threshold test for maximal electroconvulsions in mice for DRO (**a**). The drug was administered i.p., at 120 min. before electroconvulsions. Data points indicate percentage of animals with seizures (*n* = 8 mice/data point). Right panel columns illustrate median current strength values (CS_50_ in mA ± S.E.M.) for DRO (**b**), required to produce tonic hind limb extension in 50% of animals tested. Log-probit method allowed calculation of the CS_50_ values, which were statistically analyzed with one-way ANOVA followed by the post-hoc Tukey–Kramer test. *P < 0.05 and ***P < 0.001 vs. the respective control (DRO 0) group

DRO administered alone (in doses of 50, 75 and 100 mg/kg) increased, in a dose-dependent manner, the threshold for electroconvulsions in mice (Fig. 1a, b). DRO in doses of 75 and 100 mg/kg significantly elevated the threshold in experimental animals (*P* < 0.05, and *P* < 0.001, respectively, Fig. 1a, b). The CS_50_ value for DRO (50 mg/kg) did not differ significantly from the CS_50_ value for the control group (DRO 0) (Fig. 1b).

### Effect of dronedarone on the anticonvulsant potency of various classical antiepileptic drugs in the tonic–clonic seizure model in mice

**Fig. 2:**
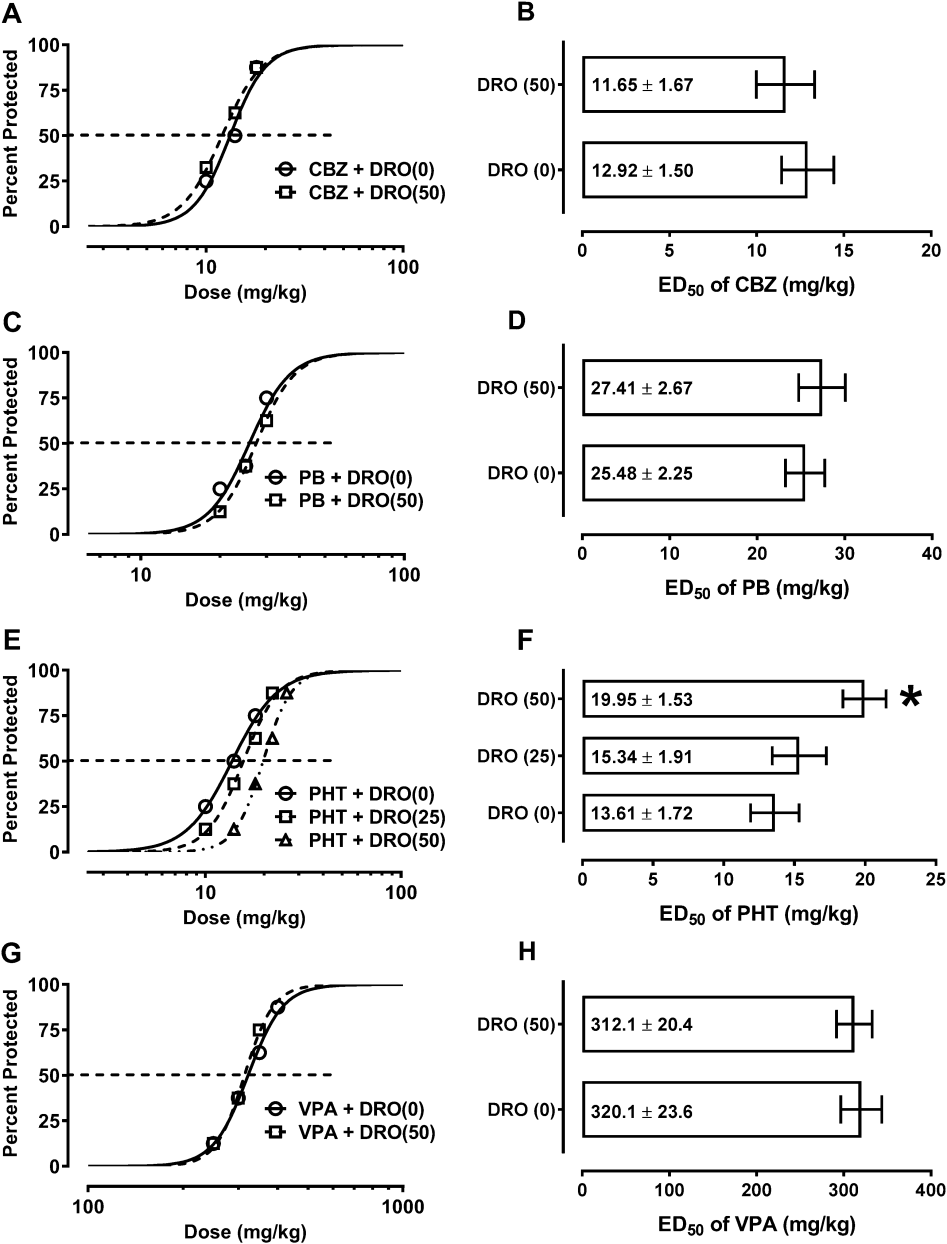
Effect of dronedarone (DRO) on the protective activity of carbamazepine (CBZ), phenobarbital (PB), phenytoin (PHT) and valproate (VPA) in the tonic–clonic seizure model in mice. Left panel graphs illustrate dose–response functions (sigmoidal curves) for the anticonvulsant activity of classical antiepileptic drugs [CBZ (**a**), PB (**c**), PHT (**e**) and VPA (**g**)] alone and in combination with DRO in the tonic–clonic seizure model. Each data point reflects percent of mice protected (*n* = 8 mice/data point) from the tonic–clonic seizure model at a given dose (in mg/kg). Points of intersections with the dashed line at 50% reflect approximate ED_50_ values of antiepileptic drugs administered alone and in combination with DRO. Right panel columns illustrate median effective doses (ED_50_ in mg/kg ± S.E.M.) of antiepileptic drugs [CBZ (**b**), PB (**d**), PHT (**f**) and VPA (**h**)] that protected half of the tested mice from the tonic–clonic seizures. The log-probit method was used to calculate the ED_50_ values. Two ED_50_ values for CBZ, PB and VPA were statistically analyzed with log-probit method. Three ED_50_ values for PHT were statistically analyzed with one-way ANOVA and post-hoc Tukey–Kramer test. *P < 0.05 vs. control (antiepileptic drug + vehicle-treated) animals

All the studied classical anticonvulsant drugs (carbamazepine, phenobarbital, phenytoin and valproate) suppressed tonic–clonic seizures in mice (Fig. 2a–h). Similarly, the combinations of the studied anticonvulsant drugs with DRO (50 mg/kg) protected the animals from electrically evoked tonic–clonic seizures. It was found that DRO (50 mg/kg) did not significantly affect the anticonvulsant potency of carbamazepine, phenobarbital and valproate in this seizure model (Fig. 2b, d, g). However, the multichannel blocker DRO considerably reduced the anticonvulsant action of phenytoin by elevating its ED_50_ value by 46% [*F* (2; 61) = 4.07, *P* = 0.022] (Fig. 2f). In contrast, DRO (25 mg/kg) had no significant effect on the anticonvulsant potency of phenytoin in the mice subjected to the tonic–clonic seizures (Fig. 2f).

### Effects of dronedarone alone and in combination with the studied antiepileptic drugs on muscular strength, motor coordination and long-term memory in mice

DRO administered alone (in a dose of 50 mg/kg) and in combination with carbamazepine, phenobarbital, phenytoin and valproate (at doses reflecting their ED_50_ values from the tonic–clonic seizure model) neither changed significantly skeletal muscular strengths, nor motor coordination in the animals subjected to the grip strength and chimney tests, respectively (Table 1). Moreover, DRO administered alone (in a dose of 50 mg/kg) and in combination with carbamazepine, phenobarbital, phenytoin and valproate did not significantly disturb learning and remembering processes in experimental animals subjected to the passive avoidance task (Table 1).

**Table 1:** Effects of dronedarone (DRO) in combinations with carbamazepine (CBZ), phenobarbital (PB), phenytoin (PHT) and valproate (VPA) on muscular strength, motor performance and long-term memory in mice

Treatment (mg/kg)	Grip strength (N)	Impaired motor coordination	Retention time (s)
Vehicle + vehicle	0.899 ± 0.057	0/8	180 (180; 180)
DRO (50) + vehicle	0.892 ± 0.059	0/8	180 (180; 180)
DRO (50) + CBZ (11.65)	0.889 ± 0.051	1/8	180 (175.7; 180)
CBZ (11.65) + vehicle	0.901 ± 0.050	0/8	180 (180; 180)
DRO (50) + PB (27.41)	0.891 ± 0.056	0/8	180 (180; 180)
PB (27.41) + vehicle	0.911 ± 0.061	0/8	180 (180; 180)
DRO (50) + PHT (19.95)	0.865 ± 0.052	1/8	180 (175.5; 180)
PHT (19.95) + vehicle	0.883 ± 0.055	0/8	180 (180; 180)
DRO (50) + VPA (312.1)	0.894 ± 0.058	1/8	180 (165.5; 180)
VPA (312.1) + vehicle	0.904 ± 0.059	0/8	180 (175; 180)

Results are presented as: (second column)—mean strengths (in newton ± S.E.M.) from the grip strength test; (third column)—number of mice with impaired motor coordination per total number of the animals in the experimental groups challenged with the chimney test; (fourth column)—median retention times (in seconds; with 25th and 75th percentiles in parentheses) from the passive avoidance task. Each experimental group comprised eight mice (for more details see Fig. 2a–h)

### Effect of dronedarone on total brain antiepileptic drug concentrations

**Fig. 3:**
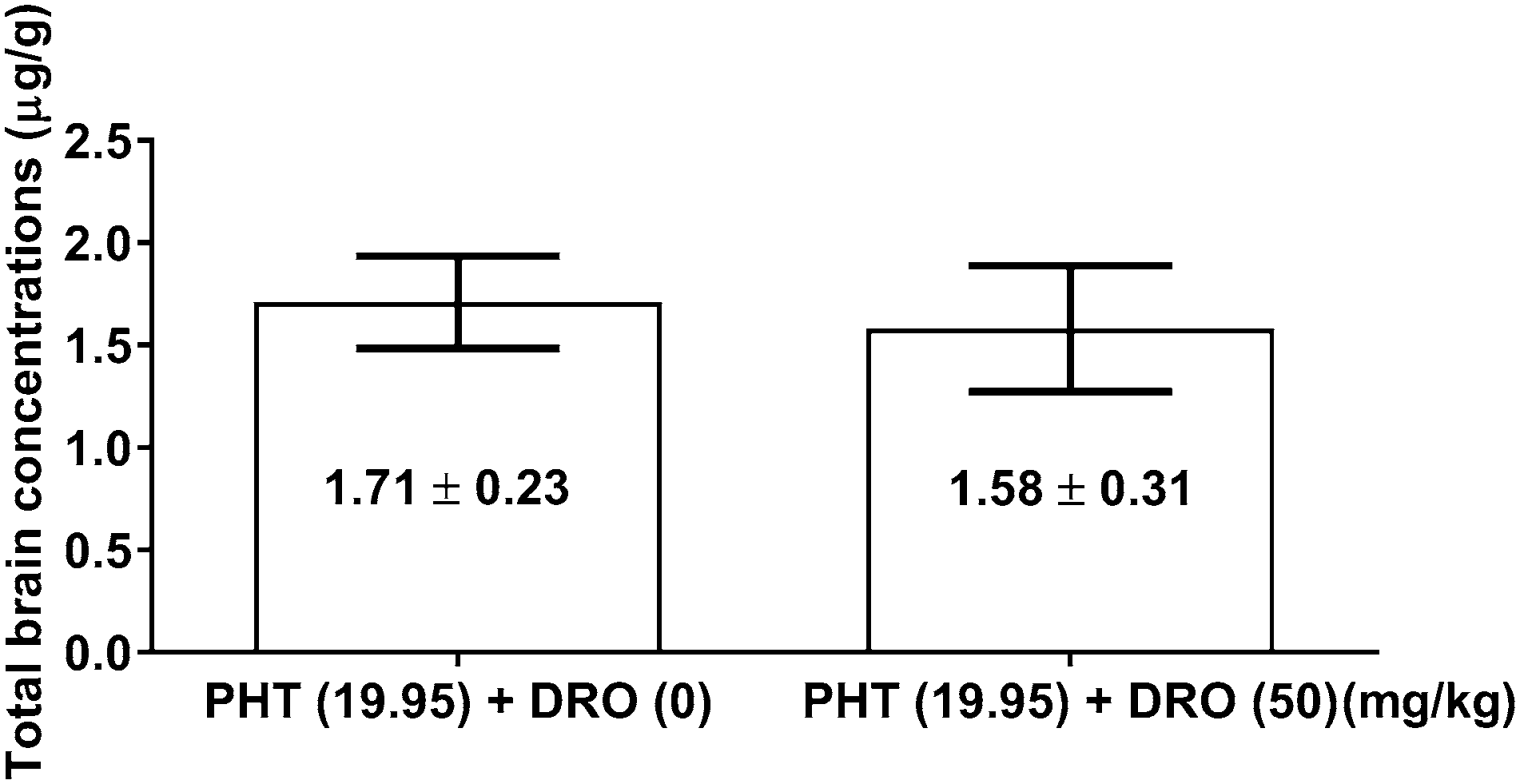
Influence of dronedarone (DRO) on total brain concentrations of phenytoin (PHT) in mice. Columns illustrate total brain concentrations (means ± S.E.M.) of PHT (*n* = 8 mice/column). No significant differences were found between both groups (the unpaired Student’s *t* test)

With fluorescence polarization immunoassay, total brain concentrations of phenytoin administered alone did not differ significantly from those determined for phenytoin in combination with DRO (Fig. 3).

## Discussion

Results presented in this study indicate that DRO, as a multichannel blocker, reduces the anticonvulsant action of phenytoin, but not that of carbamazepine, phenobarbital or valproate in the mouse tonic–clonic seizure model. The reduction of the antiseizure activity was manifested as a significant increase in the ED_50_ value for phenytoin when adding DRO, as compared to the ED_50_ value for phenytoin administered alone. Considering molecular mechanisms of action of these two drugs, it is highly likely that DRO reduces inward rapid sodium currents in neurons (Camm and Savelieva 2008; Lombardi and Terranova 2006; Savelieva and Camm 2008), whereas phenytoin binds to the inactive state of the sodium channels and blocks sodium-dependent action potentials in neurons during seizures (Czapinski et al. 2005). It seems that DRO, at least in part, blocks the site of action of phenytoin and thus, it diminishes potency of the latter drug. At present, mechanisms responsible for such a competition of both drugs are unknown, but more advanced neurochemical studies should shed some light on this phenomenon. On the other hand, DRO administered separately increases the threshold for maximal electroconvulsions and exerts by itself the antiseizure effects in mice. On the other hand, DRO did not significantly alter the anticonvulsant action of carbamazepine, whose the antiseizure activity is also associated with sodium channel blockade (Czapinski et al. 2005). Undoubtedly, there must be a distinct difference in influence of DRO on carbamazepine and phenytoin activities in the mouse maximal electroshock-induced seizure model.

In this study, we found that a significant (23%) elevation of the ED_50_ value of phenytoin in the mouse maximal electroshock-induced seizure model was accompanied with a non-significant (8%) decrease in total brain phenytoin concentrations in experimental animals. Since DRO did not significantly change total brain phenytoin concentrations, we can ascertain that interaction between DRO and phenytoin has a pharmacodynamic background related probably with a competition of DRO and phenytoin to the binding sites on sodium channels. Although this hypothesis needs verification in further neurochemical studies, it can readily explain the observed phenomenon.

As regards the other anticonvulsant drugs tested in this study, we did not evaluate their total brain concentrations because DRO did not significantly change their anticonvulsant action in the mouse maximal electroshock-induced seizure model. We did not measure total brain concentrations of phenobarbital, carbamazepine and valproate in experimental animals, therefore, we could not entirely exclude that DRO changed total brain concentrations of the rest of the investigated anticonvulsant drugs.

In this study, we also determined the potential acute adverse effects produced by the combinations of DRO with classical antiepileptic drugs. In a series of three behavioral tests, we found that DRO neither changed skeletal muscular strength in mice, nor disturbed long-term memory in mice challenged with the passive avoidance task. Additionally, DRO did not impair motor coordination in the animals subjected to the chimney test. Since DRO in combination with classical antiepileptic drugs (at doses reflecting their ED_50_ values from the maximal electroshock-induced seizure model) did not alter normal behavior in mice, the combinations produced no potential acute adverse effects in mice. When translating these results from preclinical studies to clinical settings, no acute adverse effects are expected in epilepsy patients receiving combinations of DRO with one of the classical antiepileptic drugs.

Of note, DRO can be used as an alternative drug to amiodarone, since both drugs belong to the same antiarrhythmic group of drugs (King and McGuigan 2018). Therefore, DRO can be preferentially used in patients with heart problems, who could not be treated with amiodarone. Experimental evidence indicates that amiodarone potentiated the anticonvulsant action of carbamazepine, but not that of other classical antiepileptic drugs (i.e., phenobarbital, phenytoin and valproate) (Banach et al. 2018). In contrast, we have found that DRO alleviated the anti-electroshock potency of phenytoin, but not that of carbamazepine, phenobarbital or valproate. Thus, the profile of both antiarrhythmic drugs (amiodarone and DRO) when combined with anticonvulsant drugs considerably differs. The observed interaction between phenytoin and DRO was pharmacodynamic in nature. On the other hand, by comparing the results from this study to those published earlier in similar experimental conditions, one can ascertain that DRO has a unique profile and exceptional properties. There is no doubt that molecular mechanisms of action of DRO and phenytoin should be extensively examined in further patch-clamp experiments to elucidate the mechanism(s) responsible for antagonistic interaction in mice.

Another fact should be discussed herein, when explaining pharmacokinetic estimation of total brain phenytoin content in mice. Since DRO and phenytoin were administered i.p. at 120 min before the electrically evoked seizures in this study, one could suggest that the observed reduction of the anticonvulsant effects of phenytoin in the tonic–clonic seizure model resulted from pharmacokinetic interaction related with absorption and distribution of both drugs in experimental animals. Theoretically, one drug may slow absorption and/or may diminish distribution of another drug in animals. However, we found in this study that phenytoin concentrations in the brain tissue of experimental animals (i.e., in the place of action of the drug) were almost identical in two groups of the mice, receiving phenytoin with either vehicle or DRO. Since phenytoin concentrations in the brains of experimental animals in both groups did not differ significantly, one can indirectly ascertain that DRO did not affect absorption and/or distribution of phenytoin. On the other hand, DRO and phenytoin were administered singly; therefore, pharmacokinetic changes in metabolism and elimination of the drugs were unlikely to be involved in this interaction.

Another fact must be discussed herein because phenytoin is not only the antiepileptic drug, but it is also the drug used in cardiology as a class II antiarrhythmic drug. It is highly likely that phenytoin and DRO can be used together in cardiology patients, exclusively with arrhythmias. In such a situation, a reduction of the antiarrhythmic effect of phenytoin is also expected when patients receive combined treatment with DRO and phenytoin. Of note, molecular mechanisms of action of phenytoin are not restricted to neurons, but phenytoin affects myocardial cells as well. Therefore, a similar (negative) interaction can be observed in patients receiving both drugs due to arrhythmias. Thus, a special caution is advised to patients treating with these drugs.

## Conclusions

DRO alleviates the anticonvulsant action of phenytoin in experimental animals and this interaction is pharmacodynamic in nature. Therefore, a special caution is advised when treating the patients with phenytoin and DRO. This combination of two drugs is not recommended in clinical practice and should be avoided as an unfavorable combination.

## Electronic supplementary material

Below is the link to the electronic supplementary material.

Supplementary material 1 (DOCX 14 KB)
