# Supplementary material for: Influence of dronedarone (a class III antiarrhythmic drug) on the anticonvulsant potency of four classical antiepileptic drugs in the tonic–clonic seizure model in mice
Source: J Neural Transm (Vienna). 2018 Dec 8;126(2):115–22. doi: 10.1007/s00702-018-1940-y (PMC6373245; doi:10.1007/s00702-018-1940-y)
Supplement: Supplementary file 1 — Supplementary material 1 (DOCX 14 KB) [file 702_2018_1940_MOESM1_ESM.docx]

**Supplementary Material – Table S1.** Anticonvulsant potency of carbamazepine (CBZ), phenytoin (PHT), phenobarbital (PB), valproate (VPA) alone and in combination with dronedarone (DRO) in the mouse maximal electroshock-induced seizure model.

| **Dose of CBZ (mg/kg)** | **CBZ+DRO (0)** | **CBZ+DRO (50)** |
| --- | --- | --- |
| **10** | 2/8 | 3/8 |
| **14** | 4/8 | 5/8 |
| **18** | 7/8 | 7/8 |

| **Dose of PHT (mg/kg)** | **PHT+DRO (0)** | **PHT+DRO (25)** | **PHT+DRO (50)** |
| --- | --- | --- | --- |
| **10** | 2/8 | 1/8 | - |
| **14** | 4/8 | 3/8 | 1/8 |
| **18** | 6/8 | 5/8 | 3/8 |
| **22** | - | 7/8 | 5/8 |
| **26** | - | - | 7/8 |

| **Dose of PB (mg/kg)** | **PB+DRO (0)** | **PB+DRO (50)** |
| --- | --- | --- |
| **20** | 2/8 | 1/8 |
| **25** | 3/8 | 3/8 |
| **30** | 6/8 | 5/8 |

| **Dose of VPA (mg/kg)** | **VPA+DRO (0)** | **VPA+DRO (50)** |
| --- | --- | --- |
| **250** | 1/8 | 1/8 |
| **300** | 3/8 | 3/8 |
| **350** | 5/8 | 6/8 |
| **400** | 7/8 | - |

First column represents doses of particular antiepileptic drugs used in the mouse maximal electroshock-induced seizure model. Results are presented as numbers of animals protected from tonic-clonic seizures per total numbers of animals in the experimental groups.
